# A supported palladium Schiff-base complex on SBA-15 as a reusable supported catalyst in the Heck coupling reaction

**DOI:** 10.1039/d5na00951k

**Published:** 2026-01-05

**Authors:** Amin Darabi, Mohsen Nikoorazm, Bahman Tahmasbi

**Affiliations:** a Department of Chemistry, Faculty of Science, Ilam University P. O. Box 69315516 Ilam Iran m.nikorazm@ilam.ac.ir b.tahmasbi@ilam.ac.ir

## Abstract

In this study, the mesoporous structure of SBA-15 was synthesized using a very simple procedure, and its surface was modified with 3-aminopropyltriethoxysilane (APTES). Next (3,4-bis(-(2-hydroxybenzylidene)amino)phenyl)(phenyl)methanone (bis(HBAPPM)) was obtained by condensation of salicylaldehyde (SA) and 1,2-diaminobenzophenone (diABP) in methanol (MeOH), then bis(HBAPPM) was immobilized on the modified mesoporous structure of SBA-15. Then, a palladium Schiff-base complex was supported on the functionalized SBA-15, and the final product was denoted as SBA-15@bis(HBAPPM)-Pd catalyst. The synthesized catalyst was characterized by EDX, XRD, SEM, WDX, TGA, ICP, FT-IR, and BET techniques. The catalytic application of SBA-15@bis(HBAPPM)-Pd was investigated as a heterogeneous catalyst in Heck C–C coupling reactions using various aryl halides and olefins. The result was the achievement of the desired products in excellent yields. Also, the recyclability of the SBA-15@bis(HBAPPM)-Pd nanocatalyst was studied, which showed that this catalyst can be easily isolated from the reaction medium and reused several consecutive times, which will help us in promoting green chemistry. The recovered SBA-15@bis(HBAPPM)-Pd after being reused in the reaction was characterized by EDX, XRD, SEM, WDX, ICP, and FT-IR techniques.

## Abstract 2

In this study, the mesoporous structure of SBA-15 was synthesized, and its surface was modified with APTES. Next, palladium Schiff-base complex of bis(HBAPPM) was immobilized on the modified SBA-15 as a catalyst in the Heck coupling reaction.

## Graphical abstract

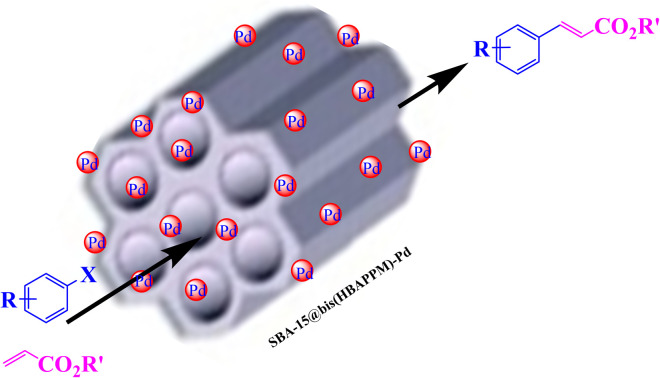

## 1. Introduction

C–C bond formation is an important and attractive class of organic coupling reactions. These reactions, which are among the most widespread synthetic transformations, have opened a new window to science and played a prominent role in various fields of science such as medicine, chemistry, biochemistry, and nanotechnology.^1–3^ Some of the best-known C–C bond formation reactions in organic chemistry include the Heck, Suzuki, Stille, Sonogashira, Kumada, Hiyama, and Negishi reactions, among others.^4–11^ Of all the cross-coupling reactions, the Heck reaction is the most important. It was first reported by Richard Heck, hence the name.^12^ Heck first reported the coupling of aryl halides (Ar-X) with ethylene in the presence of aryl mercury halides, which was a non-catalytic reaction, in 1968.^2^ Internal alkenes are important intermediates in the pharmaceutical, chemical, polymer and agricultural industries, and Mizoroki–Heck cross-coupling reactions are atom-economic routes to obtain these alkenes.^13^ Today, palladium-catalyzed Mizoroki–Heck cross-coupling reactions are an essential part of the synthesis of internal alkene derivatives and are becoming ever more complex and extensive.^14^ Nevertheless, some of the reported catalysts face the problem of difficult separation, low recyclability, and high cost of ligands.^15–18^ Traditionally, C–C bond formation is reported in organic solvents at high temperatures using homogeneous or heterogeneous Pd catalysts, including organic ligands such as phosphines, dibenzylideneacetones and sulfonates.^19^ However, organic solvents may cause serious environmental problems and high temperatures definitely incur higher costs and energy consumption. Also, most of the phosphine ligands are expensive and sensitive to air and moisture.^20,21^ Moreover, the reuse and isolation of homogeneous catalysts are difficult and environmentally unfriendly.^21^ Therefore, supported catalysts were introduced to combine the advantages of both homogeneous and heterogeneous catalysis, such as high catalytic activity, low metal loading, easily recyclable, and air- and moisture-stable catalysts as an important field in catalyst science.^22–26^ Various supports overcome most of these problems by preventing aggregation and providing an active palladium catalytic site.^15^ There are various types of supports for the design and synthesis of heterogeneous catalysts with palladium immobilization on them. Some of the supports include mesoporous silica materials,^27–29^ magnetic nanoparticles,^30^ boehmite nanoparticles,^31^ biochar nanoparticles,^32^ carbon nanotubes,^33^ graphene oxide,^34^ zeolites,^35^ and metal–organic frameworks.^36^ Mesoporous silica materials are among the latest achievements in nanotechnology. Due to their amazing chemical and physical properties, such as surface chemistry and pore architecture, these materials have many applications in various fields, *e.g.* catalysis, drug delivery, thermal energy storage, product separation, and biosensors.^37–39^ The International Union of Pure and Applied Chemistry (IUPAC) classifies porous materials into 3 categories in terms of the diameter of their pores: microporous materials (less than 2 nm), mesoporous materials (2 to 50 nm), and macroporous materials (greater than 50 nm).^40^ In recent decades, various types of mesoporous structures of Si materials have been synthesized by modifying the synthesis route and using different surfactants.^41^ The most common types of mesoporous silica include MCM (Mobil Composition of Matter), KIT (Korean Advanced Institute of Science and Technology), SBA (Santa Barbara), MSU (Michigan State University), and FDU (Fudan University).^41,42^ Among a wide range of silica-based structures, mesoporous SBA-15 has been widely investigated as a catalyst support due to its very interesting textural properties such as large and tunable pore diameters, thick walls, high surface area, large pore volume, dual pore architecture, low toxicity, dense silanol (Si–OH) groups on the surface, excellent chemical stability and versatile functionalization chemistry.^43–45^ Nanocatalysts, which are prepared by immobilizing homogeneous catalysts on solid supports, have both high efficiency and selectivity, like homogeneous catalysts.^46^ Besides, the heterogeneous and insoluble nature of nano-catalysts makes them reusable, like heterogeneous catalysts.^47,48^ Therefore, nanocatalysts do not have the limitations and disadvantages of either homogeneous or heterogeneous catalyst systems.^11^ For this reason, nanocatalysts can be called a bridge between homogeneous and heterogeneous catalysts.^49^ Therefore, in order to develop new and green methods, here we report a Schiff-base complex of palladium immobilized on mesoporous silica SBA-15 as a novel hybrid nanocatalyst. The catalytic application of this nanocatalyst was investigated and studied in the Mizoroki–Heck carbon–carbon coupling reaction.

## 2. Experimental

### 2.1. Synthesis of the bis(HBAPPM) Schiff-base ligand

**Scheme 1:**
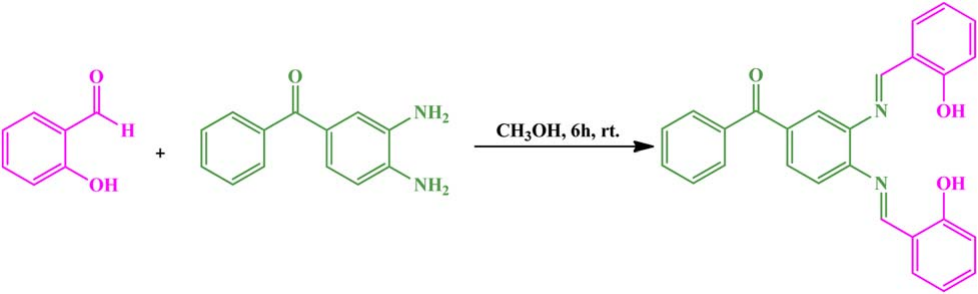
Synthesis of the (3,4-bis(-(2-hydroxybenzylidene)amino)phenyl)(phenyl)methanone Schiff-base ligand (Bis(HBAPPM)).

The (Bis(HBAPPM)) ligand was synthesized *via* a condensation reaction between SA and diABP (Scheme 1). To synthesize this Schiff-base ligand, first 4 mmol of SA was dissolved in MeOH, then 2 mmol of diABP (in MeOH) was slowly added dropwise to the solution. The reaction lasted for 6 hours at r.t. (room temperature). At the end of the reaction, an orange powder was obtained by evaporating the methanol solvent. The obtained solid product was washed with *n*-hexane and dried at r.t.^50^

### 2.2. Typical procedure for the preparation of mesoporous SBA-15

In the synthesis of the mesoporous structure of SBA-15, the nonionic and polymeric surfactant poly(ethylene glycol)-block-poly(propylene glycol)-block-poly(ethylene glycol) or P123 was used. For this purpose, first, in a 250 mL round-bottom flask, 5.34 g of surfactant P123 was added to a solution of 100 mL of HCl (0.3 M) at room temperature and completely dissolved under magnetic rotation for 12 h. Then, 8.29 g of tetraethyl orthosilicate (TEOS) was slowly injected dropwise into the reaction solution. The reaction was subjected to vigorous mixing at 35 °C for 24 h. After the desired time, the obtained solution was moved to a Teflon bottle and heated at 100 °C for 48 h. After that, the cooled mixture was separated using filter paper. The resulting white compound was dried at 100 °C for 24 h. The resulting white solid powder was extracted with an acidic ethanol solution (a mixture of 1.5 mL of hydrochloric acid and 80 mL of ethanol) for 24 h at 80 °C. To remove the surfactant pattern, the white powder was calcined at 550 °C for 5 h at a heating rate of 2 °C min^−1^^51^.

### 2.3. Modification of SBA-15 with the 3-aminopropyltriethoxysilane group

To modify the mesoporous structure of SBA-15, first, 1 g of SBA-15 was dissolved in 30 mL of *n*-hexane, then 1.5 mL of 3-aminopropyltriethoxysilane was injected into it, and the resulting mixture was heated at 60 °C for 24 h under reflux and a nitrogen atmosphere. The obtained product (SBA-15@APTES) was filtered after cooling to room temperature and washed several times with *n*-hexane, and the obtained solid was dried at r.t.

### 2.4. Functionalization of SBA-15-NPTES with the bis(HBAPPM) Schiff-base ligand

First, 1 g of SBA-15@APTES was dispersed in 30 mL of ethanol (EtOH), then 0.86 mmol of bis(HBAPPM) Schiff-base ligand was added to it. The obtained mixture was mixed using a magnetic stirrer under reflux conditions for 24 h at 80 °C. The obtained product was isolated by simple filtration and washed several times with EtOH. Finally, the solid powder (SBA-15@bis(HBAPPM)) was dried at r.t.

### 2.5. Immobilization of palladium on SBA-15 toward the preparation of the SBA-15@bis(HBAPPM)-Pd nanocatalyst

**Scheme 2:**
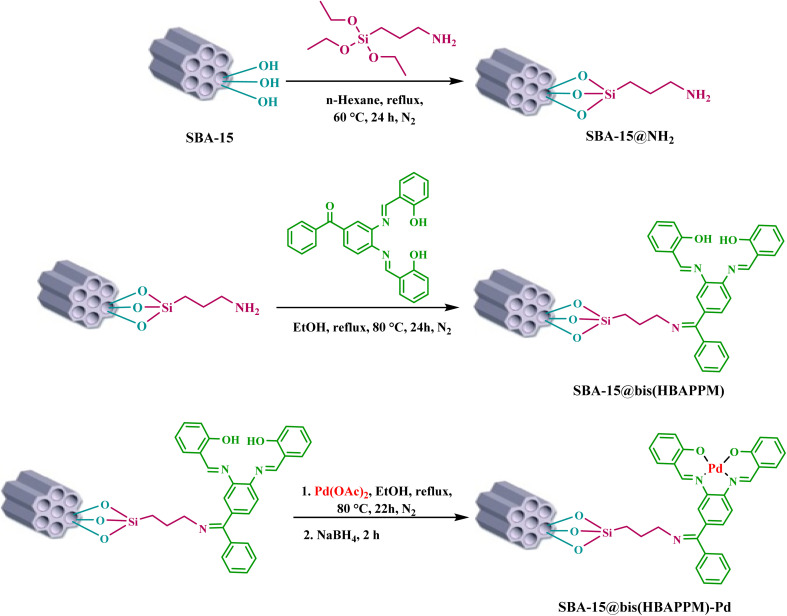
Synthesis of the SBA-15@bis(HBAPPM)-Pd nanocatalyst.

First, 1 g of functionalized SBA-15 nanoparticles (SBA-15@bis(HBAPPM)) was dispersed in 30 mL of EtOH, then 0.5 mmol of palladium acetate (Pd(OAc)_2_) was added and mixed using a magnetic stirrer under a nitrogen atmosphere at 80 °C for 22 h. After the desired time, 0.3 mmol of NaBH_4_ (sodium borohydride) was added to the reaction mixture and mixed using a magnetic stirrer under the same conditions for 2 hours. The obtained product (SBA-15@bis(HBAPPM)-Pd) was isolated after cooling, washed with EtOH and water, and finally dried at 50 °C (Scheme 2).

### 2.6. Typical method for the Mizoroki–Heck C–C cross-coupling reaction catalyzed by the SBA-15@ bis(HBAPPM)-Pd nanocatalyst

**Scheme 3:**
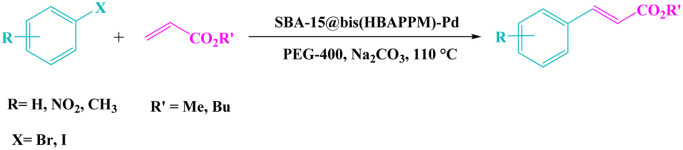
Heck reaction catalyzed by SBA-15@bis(HBAPPM)-Pd.

A mixture of SBA-15@bis(HBAPPM)-Pd nanocatalyst (0.0075 g), butyl acrylate (0.6 mmol), aryl halide (0.5 mmol), base (Na_2_CO_3_, 1.5 mmol), and 2 mL of PEG-400 (polyethylene glycol 400) as solvent was mixed using a magnetic stirrer at 110 °C. The progress of the reaction was checked using TLC. After completion of the reaction, the catalyst was removed by a paper filter, and the products were extracted with ethyl acetate. After solvent evaporation, the products with excellent yields were obtained (Scheme 3).

### 2.7. Selected spectral data

#### 2.7.1 Butyl cinnamate

^1^H NMR (250 MHz, CDCl_3_): *δ*_H_ = 7.72–7.66 (d, *J* = 15 Hz, 1H), 7.54–7.52 (d, *J* = 5 Hz, 2H), 7.39–7.37 (s, 3H), 6.48–6.42 (d, *J* = 15 Hz, 1H), 4.24–4.19 (t, *J* = 5 Hz, 2H), 1.75–1.64 (quin, *J* = 7.5 Hz, 2H), 1.49–1.37 (sex, *J* = 7.5 Hz, 2H), 1.00–0.94 (t, *J* = 7.5 Hz, 3H) ppm.

#### 2.7.2 Butyl cinnamate

^13^C NMR (100 MHz, CDCl_3_): *δ*_C_ = 167.1, 144.5, 134.5, 130.2, 128.8, 128.0, 118.3, 64.4, 30.8, 19.2, 13.7 ppm.

#### 2.7.3 Butyl (E)-3-(4-nitrophenyl)acrylate

^1^H NMR (250 MHz, CDCl_3_): *δ*_H_ = 8.26–8.23 (d, *J* = 7.5 Hz, 2H), 7.73–7.66 (m, 3H), 6.59–6.53 (d, *J* = 15 Hz, 1H), 4.25–4.20 (t, *J* = 7.5 Hz, 2H), 1.72–1.64 (quin, *J* = 7.5 Hz, 2H), 1.48–1.36 (sex, *J* = 7.5 Hz, 2H), 0.99–0.93 (t, *J* = 7.5 Hz, 3H) ppm.

## 3. Results and discussion

After the synthesis of the desired catalyst (SBA-15@bis(HBAPPM)-Pd), its structure was fully identified and confirmed using EDX, XRD, SEM, WDX, TGA, FT-IR, and BET techniques.

### 3.1. Low-angle XRD pattern

**Fig. 1:**
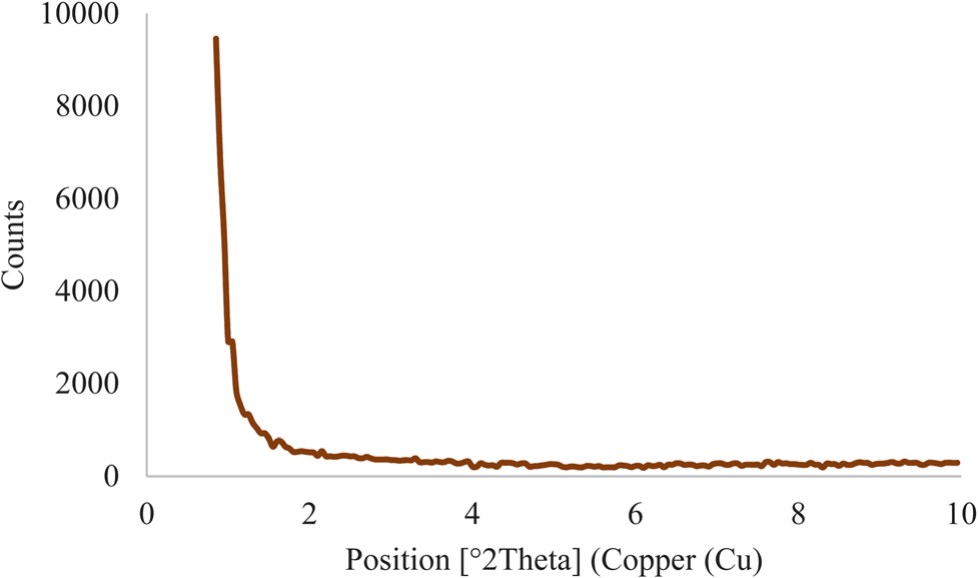
The low-angle XRD pattern of the SBA-15@bis(HBAPPM)-Pd nanocatalyst.

The X-ray diffraction technique was used to obtain more information about the structural characteristics of the SBA-15@bis(HBAPPM)-Pd nanocatalyst. The low angle XRD pattern of the SBA-15@bis(HBAPPM)-Pd nanocatalyst is shown in Fig. 1. Similar to SBA-15, the organic-inorganic hybrid material SBA-15@bis(HBAPPM)-Pd also exhibits three distinct diffraction peaks in the range of 2*θ* = 1.0, 1.50, and 1.80. These peaks correspond to reflections at *d*_100_, *d*_110_, and *d*_200_ planes, which are the indices of the highly ordered hexagonal framework of silica.^51,52^ The presence of these peaks indicates that the mesoporous crystalline order of SBA-15 in SBA-15@bis(HBAPPM)-Pd is maintained after loading. While the intensity of the peaks decreased and their angles became smaller, this indicates a decrease in mesoporous order due to the loading of SBA-15 into the pores and the expansion of the unit cell due to the binding of the palladium complex inside the pores, respectively.^53^

**Fig. 2:**
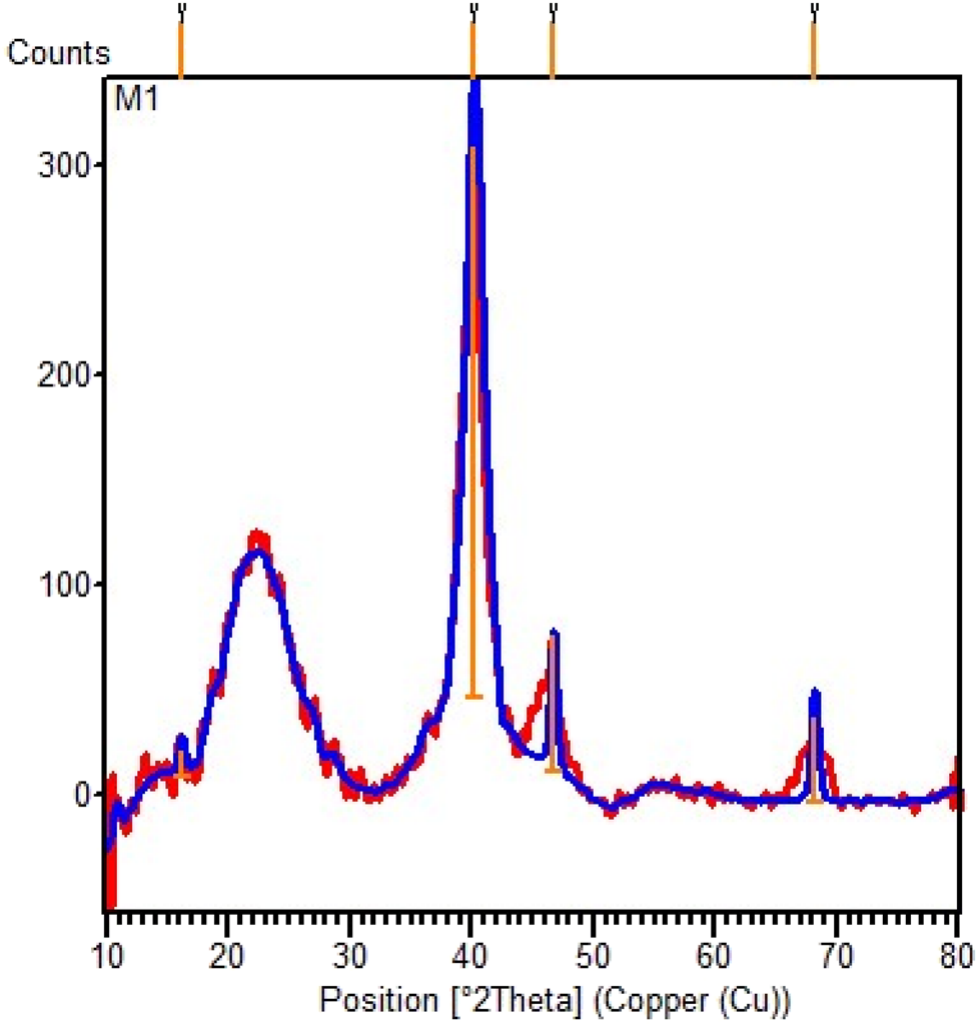
The normal XRD pattern of the SBA-15@bis(HBAPPM)-Pd nanocatalyst.

The normal XRD pattern of the SBA-15@bis(HBAPPM)-Pd nanocatalyst is shown in Fig. 2. The normal XRD pattern of the SBA-15@bis(HBAPPM)-Pd nanocatalyst exhibits three distinct diffraction peaks in the range of 2*θ* ∼40, 46, and 68. These peaks correspond to reflections at *d*_200_, *d*_220_, and *d*_331_ planes, which indicates that the chemical state of palladium in the catalyst is Pd(0).^54–56^ The broad peak in the range of 2*θ* ∼18–30 is related to amorphous silica.^56^

### 3.2. BET analysis

**Fig. 3:**
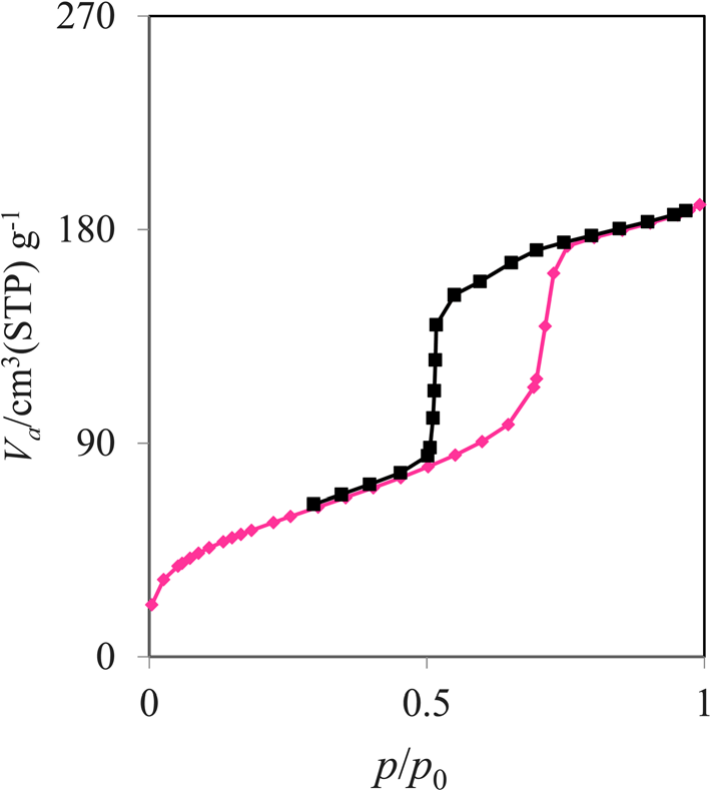
N_2_ adsorption–desorption isotherm of the SBA-15@bis(HBAPPM)-Pd nanocatalyst.

Nitrogen adsorption–desorption analysis of the synthesized SBA-15@bis(HBAPPM)-Pd nanocatalyst was carried out under standard temperature and pressure conditions. As shown in Fig. 3, this isotherm corresponds to the type IV isotherm and the H_2_ hysteresis loop according to the IUPAC classification, which is related to mesoporous materials.^57^ The values of the structural and textural parameters related to the catalyst are given in Table 1. The BET analysis data show the surface area (*S*_BET_), mean pore diameter (*r*_p_), and total pore volume parameters of the SBA-15@bis(HBAPPM)-Pd nanocatalyst to be 188.89 m^2^ g^−1^, 6.231 nm, and 0.294 cm^3^ g^−1^, respectively. These data obtained for the catalyst show lower values than those of the mesoporous SBA-15.^58^ This decrease is due to the functionalization process of SBA-15 and the placement of the Schiff-base ligand and palladium atoms within the SBA-15.

**Table 1:** The surface properties from N_2_ adsorption–desorption analysis of SBA-15 and SBA-15@bis(HBAPPM)-Pd nanocatalysts

Sample	S_BET_ (m^2^ g^−1^)	*r*_p_ (nm)	Pore volume (cm^3^ g^−1^)
SBA-15 [58]	366.0	7.2	0.71
SBA-15@ bis(HBAPPM)-Pd	188.89	6.2319	0.2943

### 3.3. SEM photographs

**Fig. 4:**
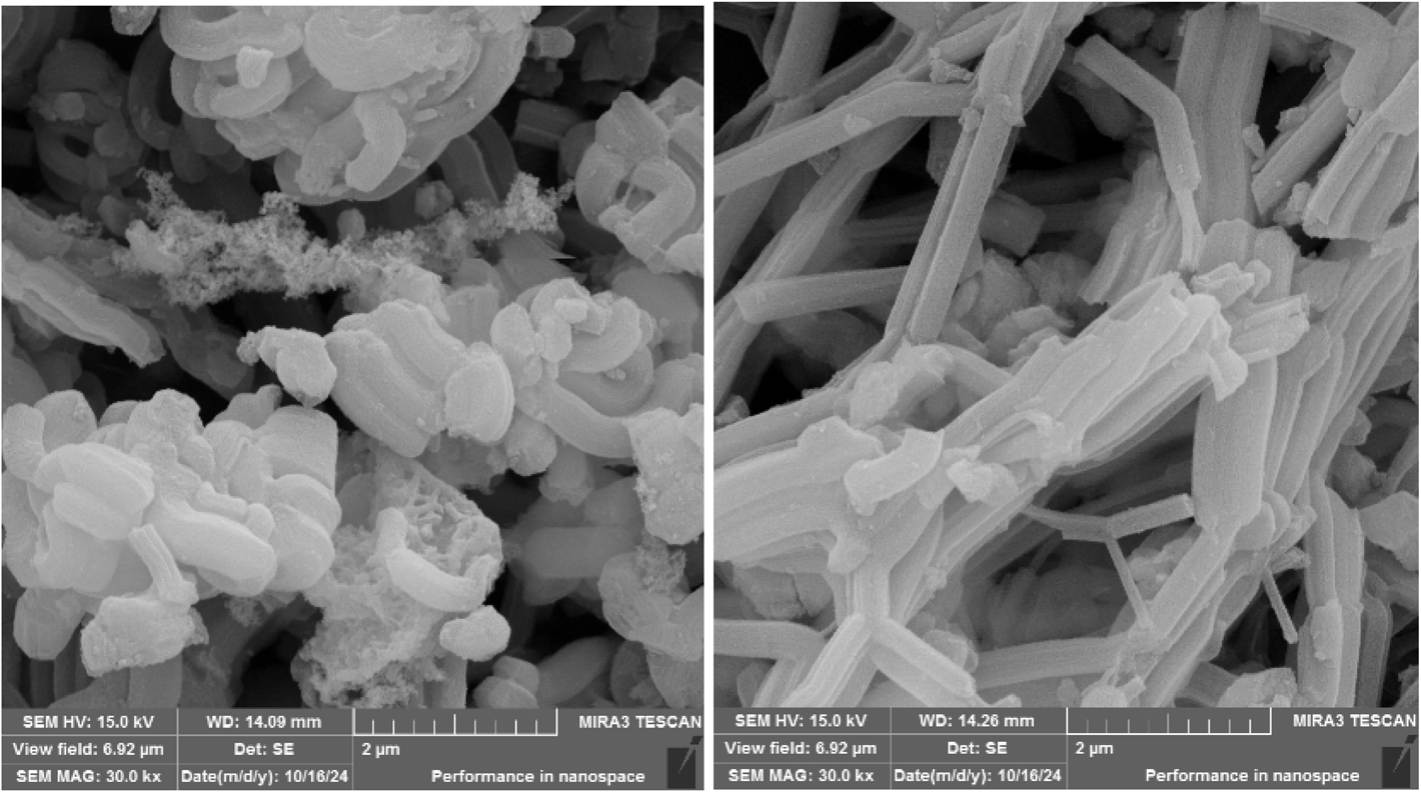
SEM images of the SBA-15@bis(HBAPPM)-Pd nanocatalyst.

Fig. 4 shows the surface of the SBA-15@bis(HBAPPM)-Pd nanocatalyst at high magnification using scanning electron microscopy (SEM). These images show that the morphology of the synthesized catalyst has a uniform particle size distribution. It also confirms the cylindrical shape of the SBA-15 mesoporous material after immobilization of the palladium complex.

### 3.4. Energy-dispersive X-ray analysis, elemental mapping and inductively coupled plasma

**Fig. 5:**
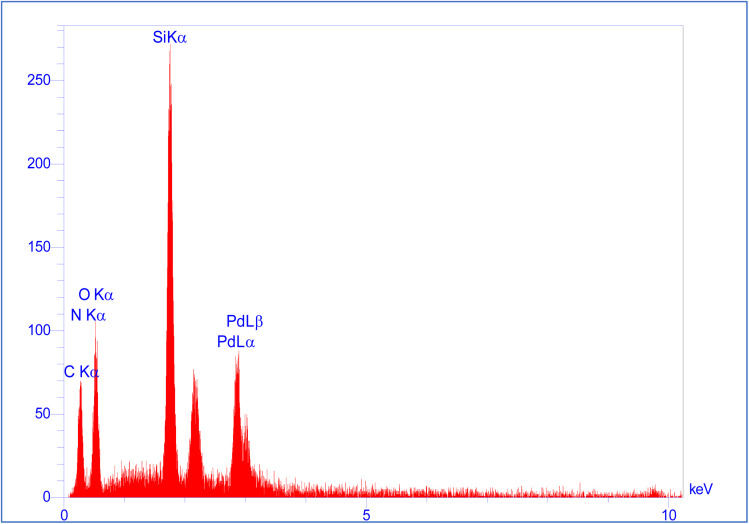
The EDX analysis of the SBA-15@bis(HBAPPM)-Pd nanocatalyst.

**Fig. 6:**
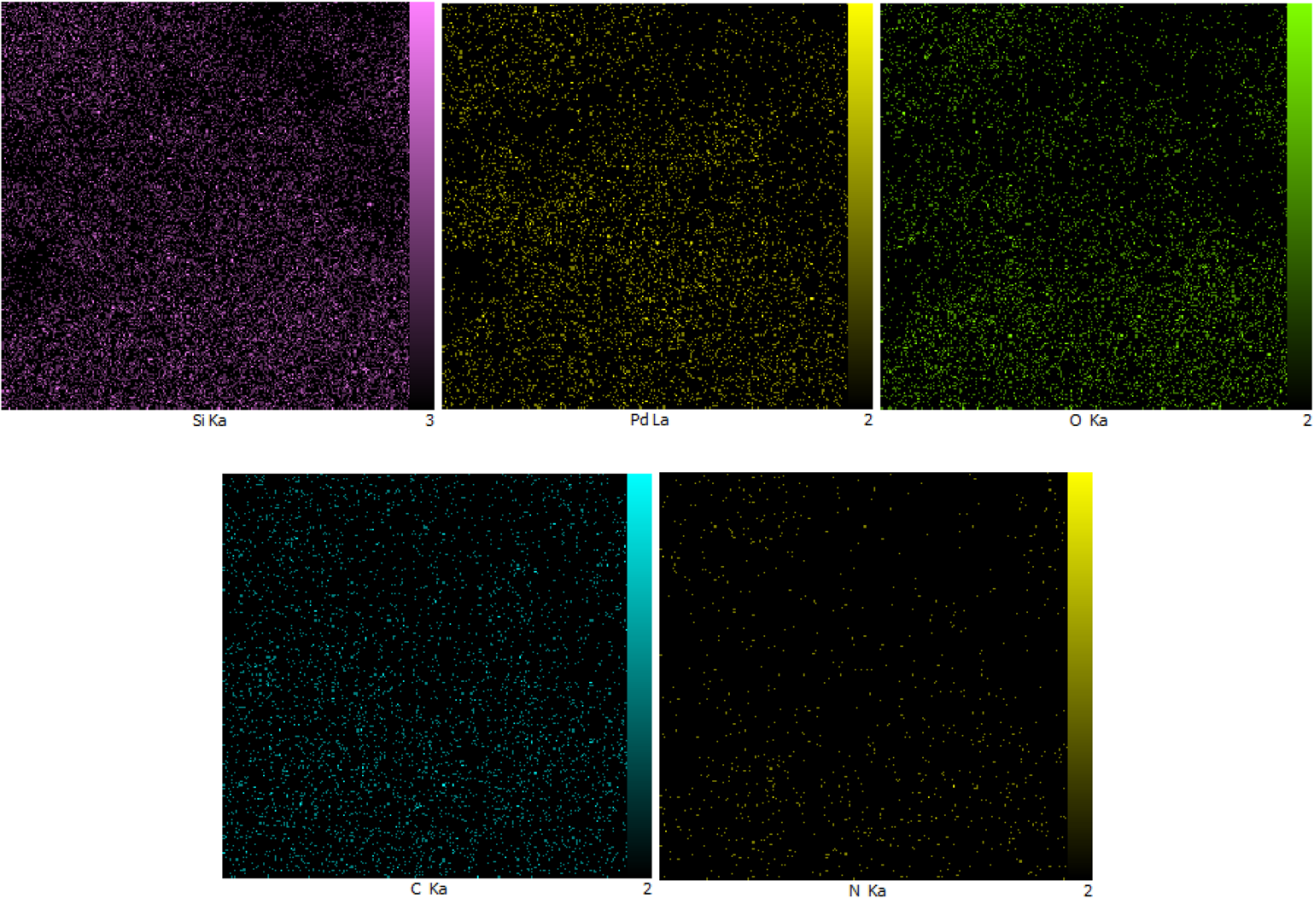
WDX elemental mapping images of the SBA-15@bis (HBAPPM)-Pd nanocatalyst.

The energy dispersive X-ray (EDX) spectrum of the SBA-15@bis(HBAPPM)-Pd nanocatalyst for determining the elemental composition is shown in Fig. 5. The EDX pattern confirms the peaks corresponding to the Si, O, C, N, and Pd species in the structure of this catalyst. Also, to determine the distribution of elements, WDX analysis was used, the images of which are shown in Fig. 6. As can be seen in the elemental mapping images, the elements Si, O, C, N, and Pd are uniformly dispersed in the structure of the SBA-15@bis(HBAPPM)-Pd nanocatalyst.

Also, the amount of palladium in the SBA-15@bis(HBAPPM)-Pd catalyst was investigated using ICP analysis. The exact amount of palladium in the SBA-15@bis(HBAPPM)-Pd catalyst was obtained as 1.4 × 10^−3^ mol g^−1^.

### 3.5. TGA

**Fig. 7:**
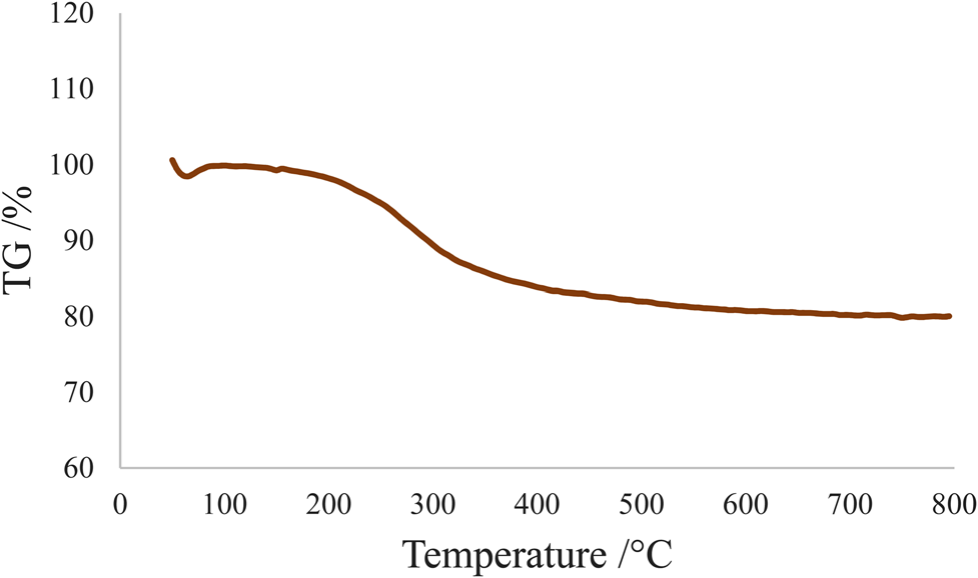
TGA curve of the SBA-15@bis (HBAPPM)-Pd nanocatalyst.

TGA was used to demonstrate the immobilization of organic groups on the SBA-15 support. The TGA curve for SBA-15@bis (HBAPPM)-Pd is shown in Fig. 7. The weight loss below 100 °C is related to the evaporation of adsorbed solvents. The weight loss of 19.34% in the temperature range of 200–500 °C is related to the thermal decomposition of organic species immobilized on the mesoporous SBA-15.^59^

### 3.6. FT-IR spectra

**Fig. 8:**
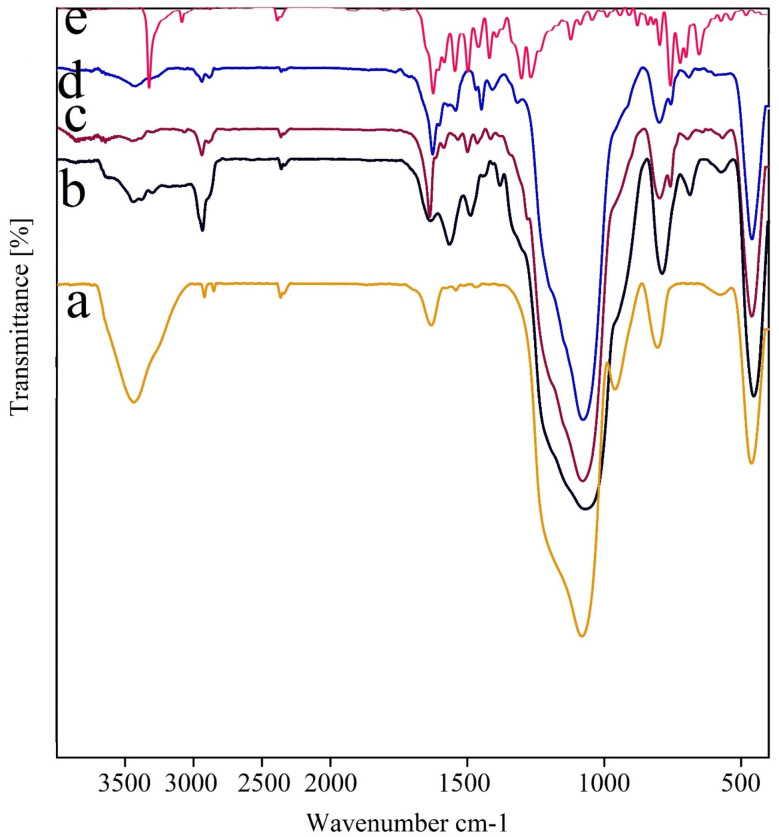
FT-IR spectra of (a) SBA-15, (b) modified SBA-15@NPTES, (c) SBA-15@ bis(HBAPPM), (d) SBA-15@bis(HBAPPM)-Pd nanocatalyst, and (e) bis(HBAPPM) ligand.

Fig. 8 illustrates the IR spectra of the synthesis steps of the nanocatalyst, including (a) SBA-15, (b) modified SBA-15@APTES, (c) SBA-15@bis(HBAPPM), (d) SBA-15@bis(HBAPPM)-Pd, and (e) bis(HBAPPM) ligand. In spectrum (a), the prominent peak at 3433 cm^−1^ is attributed to the O–H stretching vibration on the SBA-15 surface. The sharp peak at 1080 cm^−1^ is related to the asymmetric stretching vibration of the Si–O– Si, and the clear peak at 804 cm^−1^ is related to the symmetric stretching vibration of the Si–O, and the peak at 461 cm^−1^ is related to the Si–O–Si bending vibration frequency.^60,61^ In spectrum (b), which is related to the SBA-15@APTES material, the peaks related to the NH_2_ bending vibration appeared at 1492 cm^−1^ and 1567 cm^−1^, and the stretching vibration from the NH_2_ group at 3439 cm^−1^. Also, the peak related to the C–H stretching vibration was observed at 2934 cm^−1^.^62^ The presence of these peaks indicates that the modification of the SBA-15 support surface was successful. In the spectrum of SBA-15@bis(HBAPPM), a distinct peak was displayed in the 1636 cm^−1^ region, which is referred to as the stretching vibration of the imine bond C=N.^50,63^ In the FT-IR spectrum of SBA-15@bis(HBAPPM)-Pd, the presence of the bands at 461, 799, and 1077 cm^−1^ confirms that the structure of SBA-15 remains unchanged after surface modification and palladium complex immobilization.

### 3.7. Catalytic studies

To investigate the catalytic performance of SBA-15@bis(HBAPPM)-Pd in the Heck carbon–carbon coupling reaction, the reaction between butyl acrylate and iodobenzene (PhI) was selected as a model coupling reaction in the first step. At first, the model coupling reaction was examined in the presence of SBA-15 and SBA-15@bis(HBAPPM) as a catalyst (Table 2, entries 1 and 2), where no product was obtained even after 300 minutes. Therefore, the presence of palladium is essential for the Heck coupling reaction.

**Table 2:** A comparison of SBA-15@bis(HBAPPM)-Pd with SBA-15 or SBA-15@bis(HBAPPM) as a catalyst (catalyst (15 mg), butyl acrylate (1.2 mmol), iodobenzene (1 mmol), Na_2_CO_3_ (3 mmol), and PEG-400 solvent, at 110 °C)

Entry	Catalyst	Time (min)	Yield (%)
1	SBA-15	300	—
2	SBA-15@bis(HBAPPM)	300	—
3	SBA-15@bis(HBAPPM)-Pd	30	97

In the following, different reaction conditions such as the effective amount of catalyst, the effect of solvent, the effect of temperature, and the effect of the type and amount of base were tested. The results of these experiments are given in Table 3. Initially, the effect of the catalyst condensation on the sample reaction was studied in the presence of different amounts including no catalyst, 10, 12, 15, and 20 mg (Table 3, entries 1–5). The best and ideal result was achieved with an amount of 15 mg of catalyst. Reducing or increasing this amount of catalyst did not significantly improve the reaction yield. According to the results obtained, an amount of 15 mg of nanocatalyst was considered the optimal amount. In the next step, the effect of the solvent was evaluated. Different types of solvents, such as PEG-400, EtOH, DMF, toluene, DMSO, and H_2_O, were tested and investigated, and the best and ideal results were obtained in the PEG-400 solvent (Table 3, entry 4). After investigating the effect of the solvent, the effect of different bases was checked, and among the bases Na_2_CO_3_, NaOH, N(CH_2_CH_3_)_3_, K_2_CO_3_, and NaOEt, the Na_2_CO_3_ base was selected as the most effective base. Finally, the effect of temperature was tested at temperatures of 60, 90, and 110 °C. It was found that the reaction at a temperature of 110 °C has the best efficiency (Table 3, entries 4, 15, and 16).

**Table 3:** Experimental optimization of the Heck reaction of *n*-butyl acrylate and iodobenzene in the presence of the SBA-15@bis(HBAPPM)-Pd nanocatalyst^a^

Entry	Solvent	Base	Base (mmol)	Catalyst (mg)	Temperature (°C)	Time (min)	Yield (%)^b^
1	PEG-400	Na_2_CO_3_	3	—	110	300	—
2	PEG-400	Na_2_CO_3_	3	10	110	75	89
3	PEG-400	Na_2_CO_3_	3	12	110	60	90.5
**4**	**PEG-400**	**Na**_**2**_ **CO**_**3**_	**3**	**15**	**110**	**30**	**97**
5	PEG-400	Na_2_CO_3_	3	20	110	30	97
6	EtOH	Na_2_CO_3_	3	15	80	195	87
7	H_2_O	Na_2_CO_3_	3	15	100	420	87
8	DMF	Na_2_CO_3_	3	15	110	90	91
9	DMSO	Na_2_CO_3_	3	15	110	180	—
10	Toluene	Na_2_CO_3_	3	15	110	180	—
11	PEG-400	NaOEt	3	15	110	90	88
12	PEG-400	NaOH	3	15	110	210	90
13	PEG-400	N(CH_2_CH_3_)_3_	3	15	110	120	89
14	PEG-400	K_2_CO_3_	3	15	110	120	92
15	PEG-400	Na_2_CO_3_	3	15	90	90	89
16	PEG-400	Na_2_CO_3_	3	15	60	240	80
17	PEG-400	Na_2_CO_3_	2	15	110	80	91
18	PEG-400	Na_2_CO_3_	1	15	110	120	90

a Reaction conditions: SBA-15@bis(HBAPPM)-Pd nanocatalyst (0–20 mg), butyl acrylate (1.2 mmol), iodobenzene (1 mmol), base (1–3 mmol), and solvent (2 mL), at 60–110 °C.

b Isolated yield.

In summary, the results in the table show that the conditions selected for carrying out the Heck reaction are the use of Ar-X (1 mmol) with butylacrylate (1.2 mmol) in the presence of 15 mg of SBA-15@bis(HBAPPM)-Pd catalyst, 3 mmol of Na_2_CO_3_ base, in PEG-400 solvent and at a temperature of 110 °C (Table 3, entry 4).

The catalytic application of SBA-15@bis(HBAPPM)-Pd in the synthesis of a wide range of methyl cinnamate or butyl cinnamate derivatives was tested on a number of different aryl halides under the optimized conditions, which are summarized in Table 4. The results of these experiments are summarized in Table 4, which shows that the desired methyl cinnamate or butyl cinnamates were synthesized in good yields. TOF and TON values indicate that aryl halides with electron-withdrawing groups are more reactive than aryl halides with electron-donating groups in the Heck carbon–carbon coupling reaction in the presence of the SBA-15@bis(HBAPPM)-Pd catalyst.

**Table 4:** Heck reaction in the presence of the SBA-15@bis(HBAPPM)-Pd nanocatalyst^a^

Entry	X	R	R′	Time (min)	Yield^b^ (%)	TON	TOF (h^−1^)
1	I	H	Bu	30	97	46.19	92.38
2	Br	H	Bu	40	94	44.76	67.14
3	Br	4-NO_2_	Bu	30	96	45.71	91.43
4	I	4-NO_2_	Bu	30	94	44.76	89.52
5	I	H	Me	60	95	45.24	45.24
6	Br	H	Me	85	95	45.24	31.93
7	I	4-CH_3_	Me	65	94	44.76	41.32
8	Br	4-NO_2_	Me	95	93	44.28	27.97
9	Br	4-CH_3_	Me	95	95	45.24	28.57
10	I	4-NO_2_	Me	50	94	44.76	53.71

a Reaction conditions: SBA-15@bis(HBAPPM)-Pd nanocatalyst (15 mg), methyl acrylate or butyl acrylate (1.2 mmol), aryl halide (1 mmol), Na_2_CO_3_ (3 mmol), and PEG-400 solvent, at 110 °C.

b Isolated yield.

**Scheme 4:**
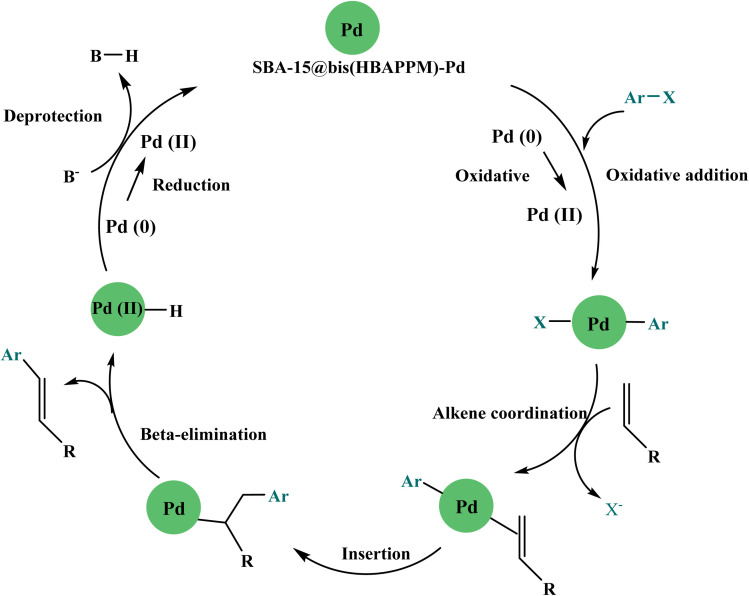
Proposed mechanism for the Heck carbon–carbon coupling reaction in the presence of the SBA-15@bis(HBAPPM)-Pd nanocatalyst.

The proposed catalytic mechanism for the Heck carbon–carbon coupling reaction catalyzed by SBA-15@bis(HBAPPM)-Pd is presented in Scheme 4. The cycle consists of an (1) oxidative addition, (2) an insertion step, (3) a beta-hydride elimination step, and (4) a reductive elimination step, which at the end of the catalysis is recycled and returned to the cycle.^54,64–68^ In the oxidative addition step, the Pd(0) is converted to Pd(ii) after the addition of the aryl halide. In the insertion step, an olefinic species is added, and a new C–C bond is formed. In the β-hydride elimination step, the H atom migrates, and palladium hydride and products are formed. Finally, in the reductive elimination step, the Pd(0) is regenerated by a reduction reaction.^69^

### 3.8. Reusability of the SBA-15@bis (HBAPPM)-Pd catalyst

**Fig. 9:**
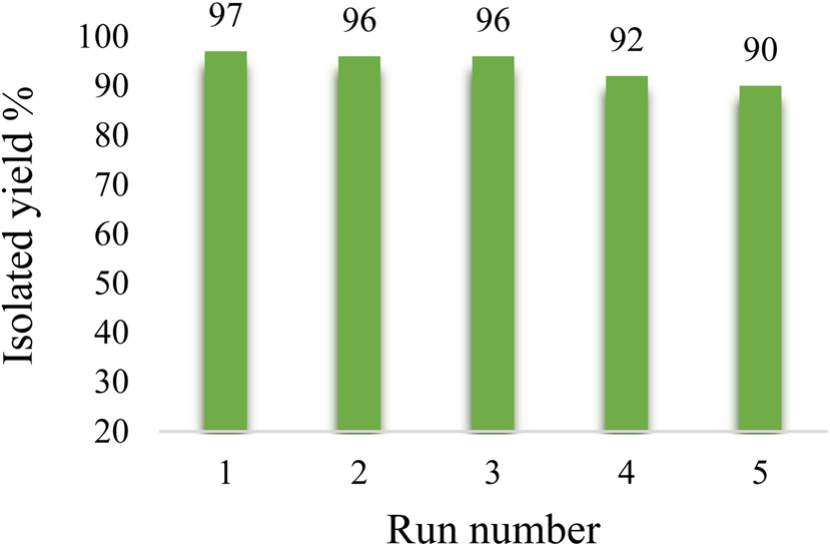
The reusability of the SBA-15@bis(HBAPPM)-Pd nanocatalyst in the model reaction of butyl cinnamate synthesis.

Recyclability is the most important feature of catalytic systems. The SBA-15@bis(HBAPPM)-Pd catalyst is a heterogeneous catalyst that is easily separated from the product after reaction by simple filtration. Hence, it can be easily used to carry out the reaction again. This is proof of the value and greenness of a catalyst. Therefore, the reaction between PhI and butyl acrylate under the optimal conditions in Table 3 was considered as a model reaction to start recycling. After reaction, the SBA-15@bis(HBAPPM)-Pd catalyst was isolated from the reaction mixture by filter paper and thoroughly washed using ethyl acetate solvent. After drying at 50 °C, it was used for reuse under the same conditions. As shown in Fig. 9, the recycling of the SBA-15@bis(HBAPPM)-Pd catalyst is repeated several times. This catalyst, with almost constant catalytic activity, can be recycled up to 5 times. The excellent reusability may result from the strong bond between the palladium complex and the SBA-15 support, which effectively prevents palladium leaching.

The stability and heterogeneous nature of SBA-15@bis(HBAPPM)-Pd were investigated by a hot filtration test based on a published article.^70^ In this regard, in the synthesis of butyl cinnamate from the coupling of iodobenzene with butyl acrylate, the catalyst was removed after 15 min, and the remaining mixture was allowed to react for 30 min without the catalyst. In this hot filtration test, the butyl cinnamate product was obtained in 67% yield. These results indicate that no significant leaching of palladium occurred during the reaction. Also, the coupling reaction of iodobenzene with butyl acrylate was repeated, and after 30 minutes, the SBA-15@bis(HBAPPM)-Pd catalyst was isolated by simple filtration. The amount of palladium in the filtered solution and the recovered SBA-15@bis(HBAPPM)-Pd catalyst was determined using ICP analysis. No leached palladium was observed in the filtered solution. Also, the exact amount of palladium in the recovered SBA-15@bis(HBAPPM)-Pd catalyst was obtained as 1.2 × 10^−3^ mol g^−1^, which does not change significantly compared to the palladium present in the fresh catalyst (1.4 × 10^−3^ mol g^−1^). Therefore, it can be said with certainty that palladium leaching from the catalyst did not occur under the Heck C–C coupling reaction conditions.

### 3.9. The post-recovery characterization of the SBA-15@bis(HBAPPM)-Pd catalyst

The post-recovered SBA-15@bis(HBAPPM)-Pd nanocatalyst was characterized by EDX, XRD, SEM, WDX, ICP, and FT-IR techniques, and was matched with a fresh catalyst.

**Fig. 10:**
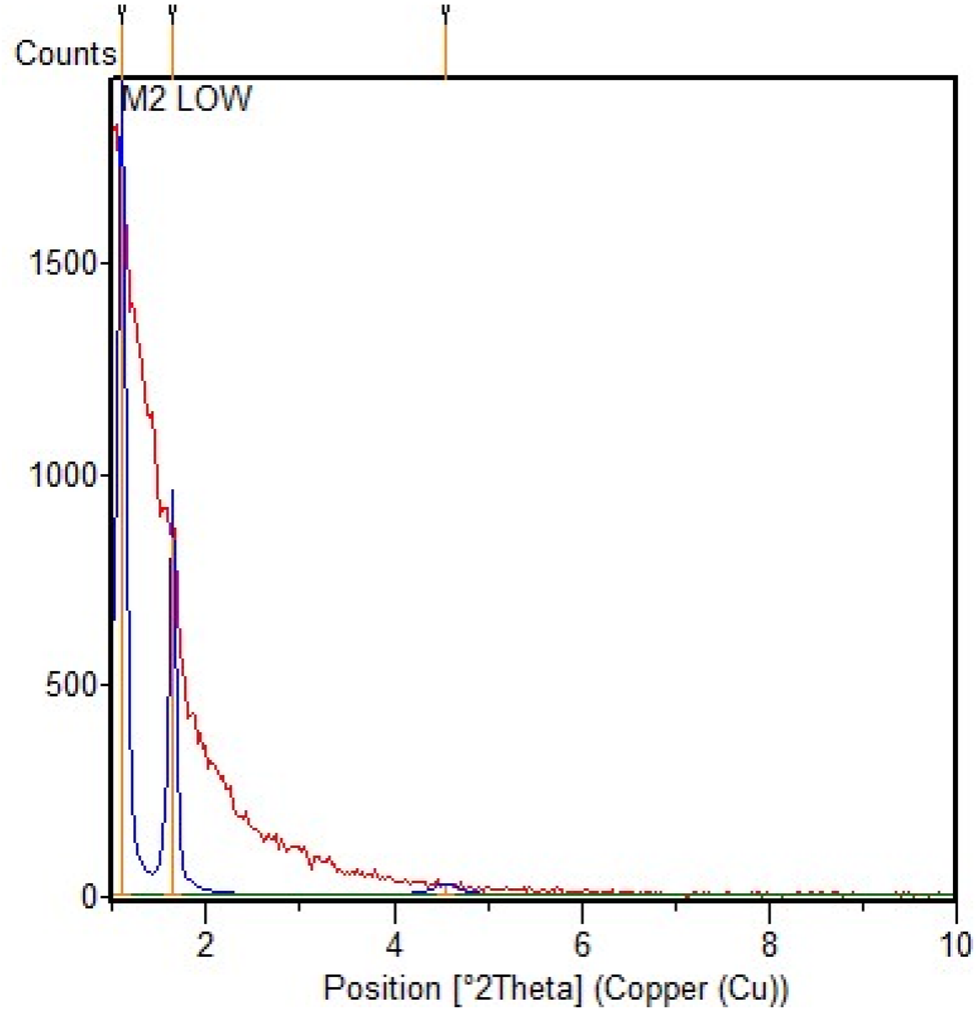
The low-angle XRD pattern of the post-recovered SBA-15@bis(HBAPPM)-Pd nanocatalyst.

The low-angle XRD pattern of the post-recovered SBA-15@bis(HBAPPM)-Pd nanocatalyst is shown in Fig. 10. Similar to fresh SBA-15@bis(HBAPPM)-Pd, the low-angle XRD pattern of the post-recovered catalyst shows three distinct 2*θ* diffraction peaks, which are indicators of the stability of this catalyst.

**Fig. 11:**
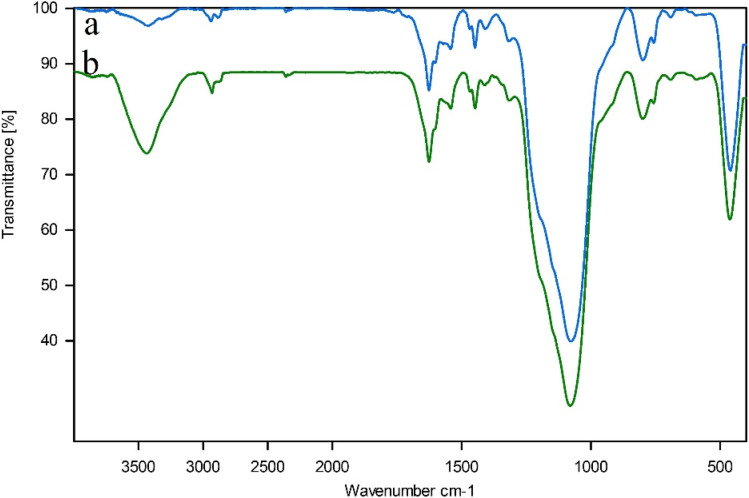
FT-IR spectra of (a) the SBA-15@bis(HBAPPM)-Pd nanocatalyst and (b) the post-recovered SBA-15@bis(HBAPPM)-Pd nanocatalyst.

Fig. 11 illustrates the IR spectra of the fresh SBA-15@bis(HBAPPM)-Pd and post-recovered SBA-15@bis(HBAPPM)-Pd. The IR spectrum of the post-recovered catalyst is in perfect agreement with the IR spectrum of the fresh catalyst, indicating the stability of the catalyst under the reaction conditions after recovery.

**Fig. 12:**
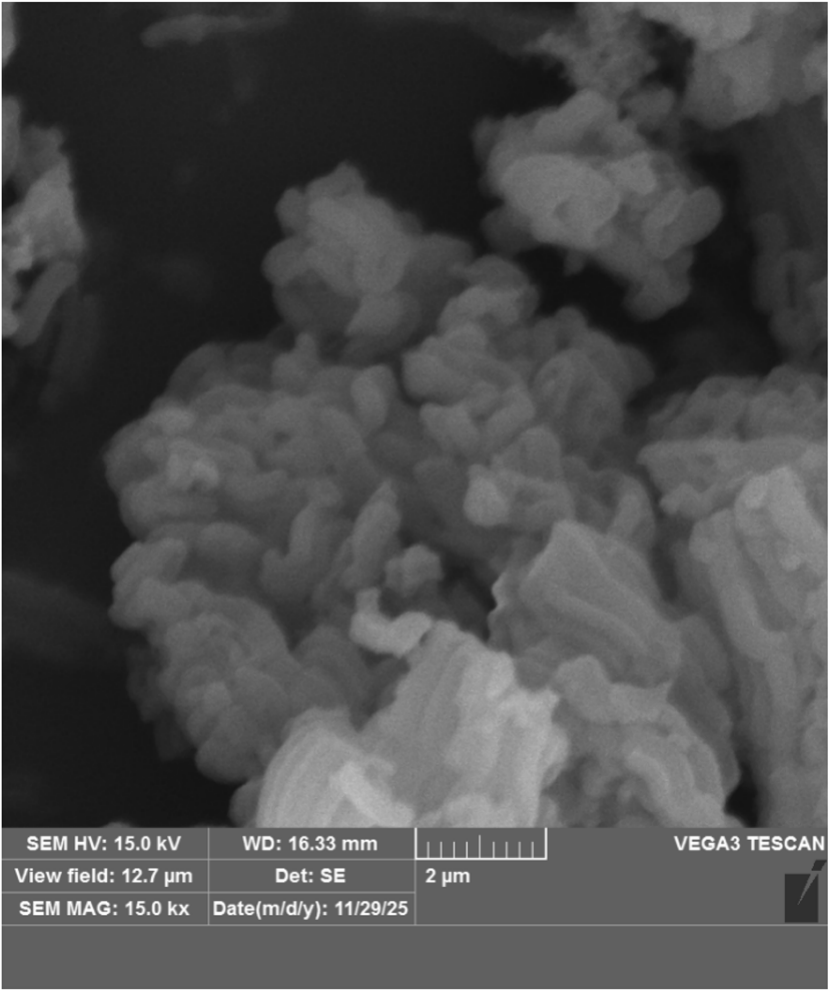
SEM images of the post-recovered SBA-15@bis(HBAPPM)-Pd nanocatalyst.

Fig. 12 shows the SEM image of the post-recovered SBA-15@bis(HBAPPM)-Pd nanocatalyst. This image shows that the morphology of the post-recovered catalyst has a uniform particle size distribution, similar to that of the fresh catalyst. The morphology and particle size of the catalyst remained unchanged after recycling, indicating the stability of the catalyst after recycling.

**Fig. 13:**
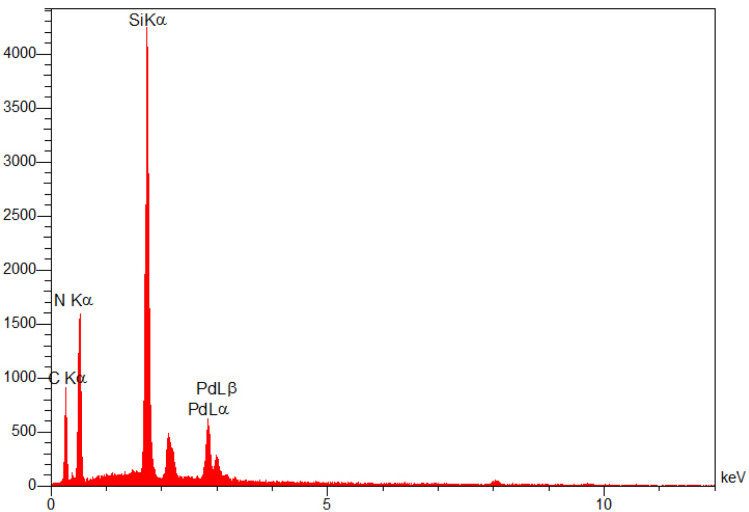
EDX analysis of the post-recovered SBA-15@bis(HBAPPM)-Pd nanocatalyst.

**Fig. 14:**
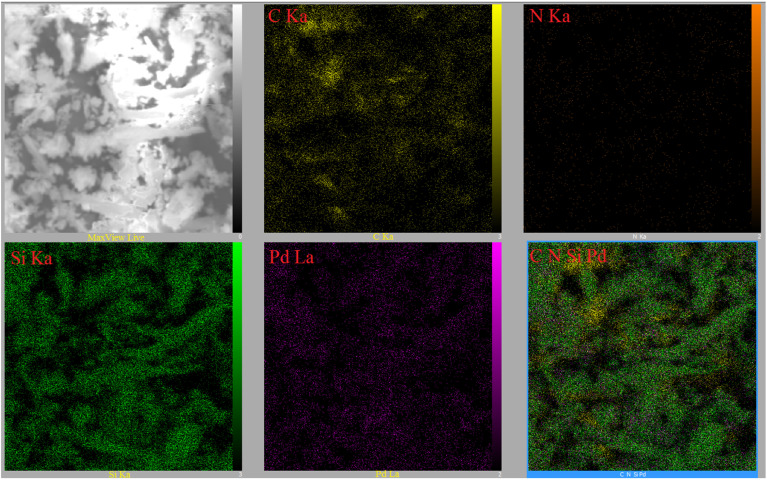
WDX elemental mapping images of the post-recovered SBA-15@bis (HBAPPM)-Pd nanocatalyst.

The EDX spectrum and WDX mapping of the post-recovered SBA-15@bis(HBAPPM)-Pd catalyst are shown in Fig. 13 and 14. The EDX pattern of the post-recovered catalyst (Fig. 13) confirms the presence of the Si, O, C, N, and Pd species, similar to that of the fresh catalyst. Also, WDX analysis of the post-recovered catalyst (Fig. 14) shows that all elements are uniformly dispersed in the structure of the recovered nanocatalyst.

Also, the amount of palladium in the post-recovered SBA-15@bis(HBAPPM)-Pd catalyst was calculated as 1.2 × 10^−3^ mol g^−1^, which does not change significantly compared to the palladium present in the fresh catalyst (1.4 × 10^−3^ mol g^−1^). Therefore, palladium leaching from the catalyst did not occur under the Heck C–C coupling reaction conditions.

### 3.10. Comparison of the catalyst

Table 5 compares the performance and activity of the SBA-15@bis(HBAPPM)-Pd catalyst with previous catalysts reported for this reaction. The comparison was made for the reaction of PhI with butyl acrylate. As is clear, this reaction was carried out using the SBA-15@bis(HBAPPM)-Pd catalyst in the green solvent PEG-400 in a shorter time with high efficiency. In addition, the SBA-15 support is a cheap and environmentally friendly support. Also, SBA-15@bis(HBAPPM)-Pd is a heterogeneous catalyst and is easily separated from the reaction medium. In some cases, toxic solvents, expensive catalysts, or homogeneous and non-recyclable catalysts have been used, which are difficult to separate from the reaction medium.

**Table 5:** Comparison of the SBA-15@bis(HBAPPM)-Pd nanocatalyst for the Heck reaction with reported catalysts in the literature

Entry	Reported catalysts	Reaction conditions	Time (min)	Yield (%)	Ref.
1	boehmite@tryptophan-Pd	DMSO, K_2_CO_3_, 120 °C	85	95	71
2	f-CNTs-Pd	DMF, Et_3_N, 100 °C	7.5 h	64.2	72
3	Pd@NHC-MOP	DMF, Et_3_N, 130 °C	60	99	73
4	C-(KTB-Pd)	DMF, K_3_PO_4_·3H_2_O, 120 °C	120	99	74
5	Pd(ii)-SBA-16	H_2_O, K_2_CO_3_, 80 °C	60	97	75
6	Pd/SBA-15	DMF, Et3N, 100 °C	12 h	88	76
7	Bis(oxamato)palladate(II)	DMF, Et_3_N, 80 °C	180	94	77
8	SBA-15@bis(HBAPPM)-Pd	PEG-400, Na_2_CO_3_, 110 °C	30	97	Current study

## 4. Conclusion

SBA-15 nanoparticles are used as a suitable support for stabilizing metal complexes and producing heterogeneous nanocatalysts, which, in addition to increasing catalytic activity, allow for convenient and easy separation of metal complexes. In this study, SBA-15@bis(HBAPPM)-Pd, as a new nanocatalyst, was first synthesized by immobilization of a palladium complex in the mesoporous channels of SBA-15, then characterized by EDX, XRD, SEM, WDX, TGA, FT-IR, and BET techniques. The prepared catalyst was used in the Mizoroki–Heck carbon–carbon coupling reaction, which is known as one of the important reactions in organic chemistry. The carbon–carbon coupling reaction is important in the synthesis of biologically active compounds, organic building blocks, natural synthetic compounds, and pharmaceutical compounds and intermediates. The advantages of this catalytic system include minimal time and cost, no waste generation, production of products with good yields and short reaction times, use of green solvents, simplicity of operation, ease of catalyst recovery, and good performance under reaction conditions. In addition, SBA-15@bis(HBAPPM)-Pd is economically viable and environmentally friendly due to the negligible Pd washout.

## Author contributions

Amin Darabi: methodology, validation, investigation, writing – original draft. Bahman Tahmasbi: conceptualization, resources, writing – original draft, project administration. Mohsen Nikoorazm: formal analysis, resources, writing – review & editing, project administration.

## Conflicts of interest

Authors declare no conflict of interest.

## Supplementary Material

NA-008-D5NA00951K-s001

## Data Availability

All data generated or analyzed during this study are included in this published article and supporting information (SI). Supplementary information is available. See DOI: https://doi.org/10.1039/d5na00951k.
