# Supplementary material for: A supported palladium Schiff-base complex on SBA-15 as a reusable supported catalyst in the Heck coupling reaction
Source: Nanoscale Adv. 2026 Jan 5;8(5):1712–24. doi: 10.1039/d5na00951k (PMC12892098; doi:10.1039/d5na00951k)

Supplementary Information (SI) for Nanoscale Advances.
This journal is © The Royal Society of Chemistry 2026

Supplementary Data

A Supported palladium Schiff-base complex on SBA-15 as reusable supported catalyst in the Heck coupling reaction

Amin darabi | Mohsen Nikoorazm* | Bahman Tahmasbi*

Abstract

In this study, the mesoporous structure of SBA-15 was synthesized using a very simple procedure, and its surface was modified with 3-aminopropyltriethoxysilane (APTES). Next, (3,4-bis(-(2-hydroxybenzylidene)amino)phenyl)(phenyl)methanone (bis(HBAPPM)) was obtained by condensation of salicylaldehyde (SA) and 1,2-diaminobenzophenone (diABP) in methanol (MeOH), then bis(HBAPPM) was immobilized on the modified mesoporous structure of SBA-15. Then, a palladium Schiff-base complex was supported on the functionalized SBA-15, and the final product was denoted as SBA-15@bis(HBAPPM)-Pd catalyst. The synthesized catalyst was characterized by EDX, XRD, SEM, WDX, TGA, ICP, FT-IR, and BET techniques. The catalytic application of SBA-15@bis(HBAPPM)-Pd was investigated as a heterogeneous catalyst in Heck C – C coupling reaction using various aryl halides and olefins. The result was the achievement of the desired products with excellent yields. Also, the recyclability of the SBA-15@bis(HBAPPM)-Pd nanocatalyst was studied, which showed that this catalyst can be easily isolated from the reaction medium and reused for several consecutive times, which will help us in promoting green chemistry. The recovered SBA-15@bis(HBAPPM)-Pd after reusing from the reaction was characterized by EDX, XRD, SEM, WDX, ICP, and FT-IR techniques.

Keywords: Mesoporous SBA-15, nanocatalyst, palladium, Schiff-base complex, Mizoroki-Heck reaction.

^{1}H NMR spectral data

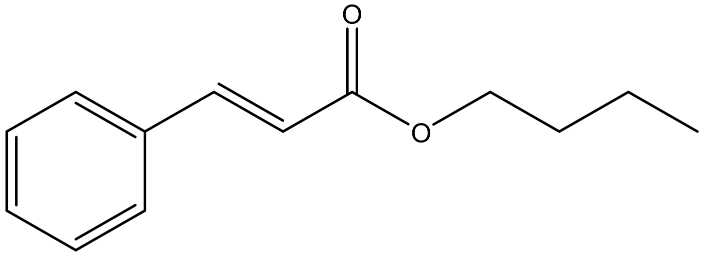

butyl cinnamate

^{1}H NMR (250 MHz, $CDCl_3$): δ_H= 7.72-7.66 (d, *J*= 15 Hz, 1H), 7.54-7.52 (d, *J*= 5 Hz, 2H), 7.39-7.37 (s, 3H), 6.48-6.42 (d, *J*= 15 Hz, 1H), 4.24-4.19 (t, *J*= 5 Hz, 2H), 1.75-1.64 (quin, *J*= 7.5 Hz, 2H), 1.49-1.37 (sex, *J*= 7.5 Hz, 2H), 1.00-0.94 (t, *J*= 7.5 Hz, 3H) ppm.

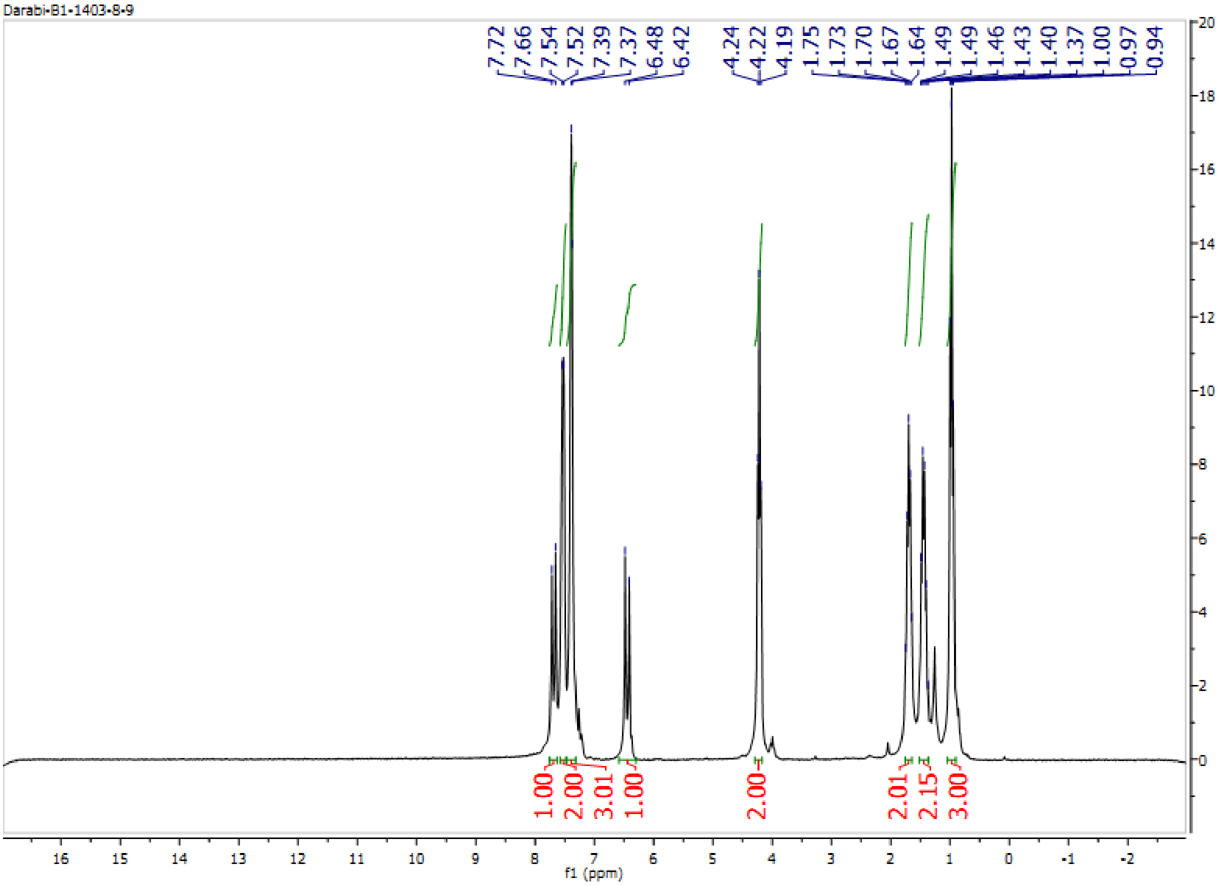

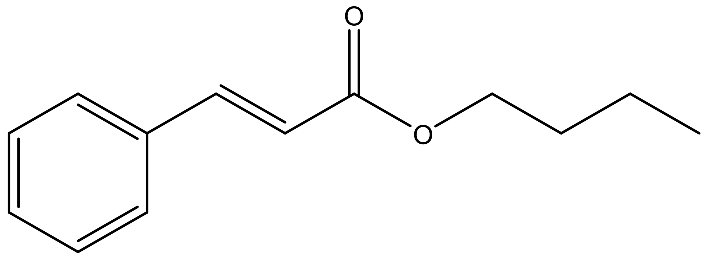

butyl cinnamate

^{13}C NMR (100 MHz, $CDCl_3$): δ_C= 167.1, 144.5, 134.5, 130.2, 128.8, 128.0, 118.3, 64.4, 30.8, 19.2, 13.7 ppm.

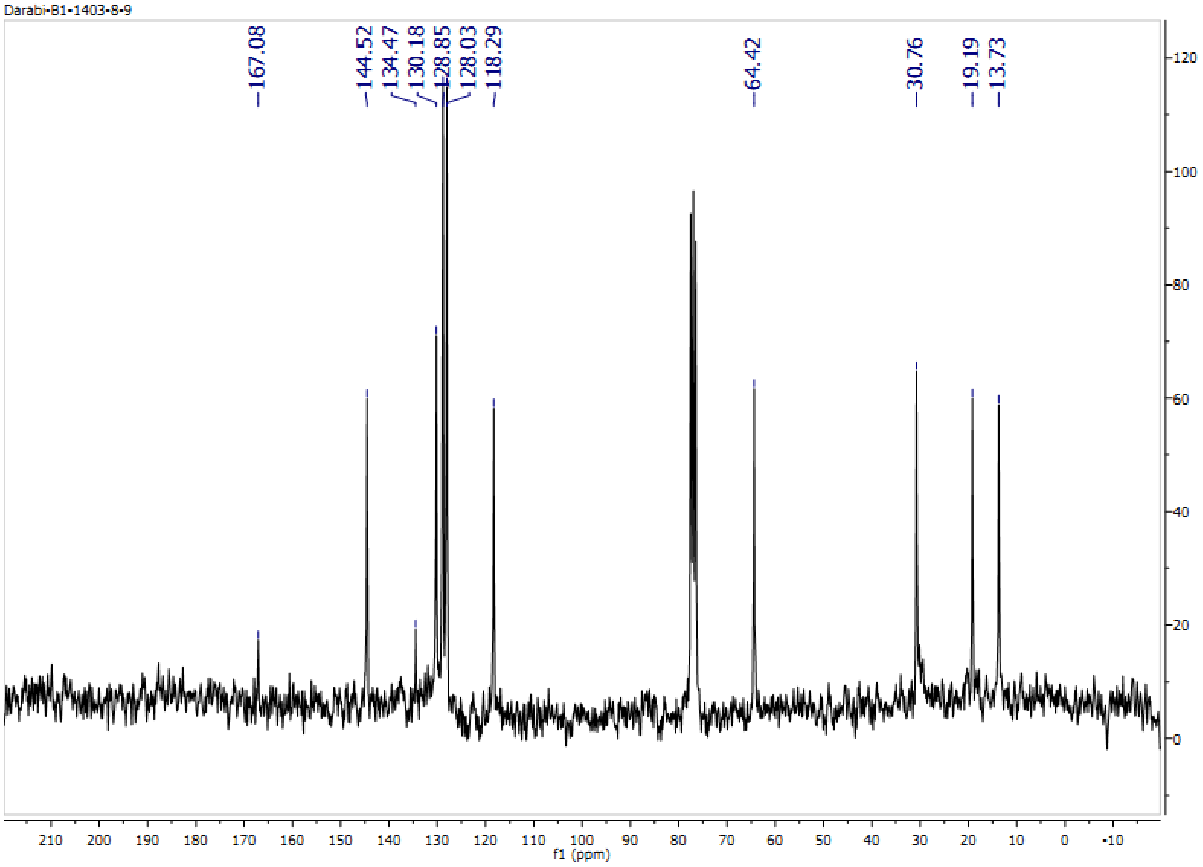

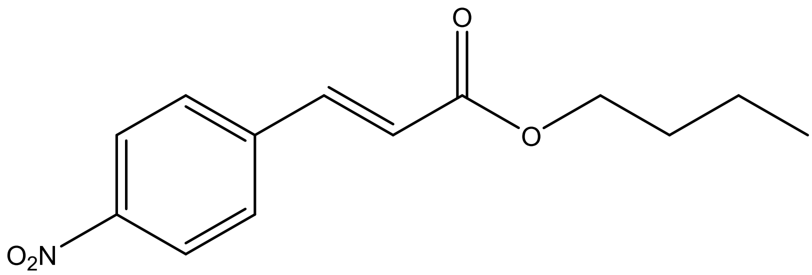

butyl (E)-3-(4-nitrophenyl)acrylate

^{1}H NMR (250 MHz, $CDCl_3$): δ_H= 8.26-8.23 (d, *J*= 7.5 Hz, 2H), 7.73-7.66 (m, 3H), 6.59-6.53 (d, *J*= 15 Hz, 1H), 4.25-4.20 (t, *J*= 7.5 Hz, 2H), 1.72-1.64 (quin, *J*= 7.5 Hz, 2H), 1.48-1.36 (sex, *J*= 7.5 Hz, 2H), 0.99-0.93 (t, *J*= 7.5 Hz, 3H) ppm.

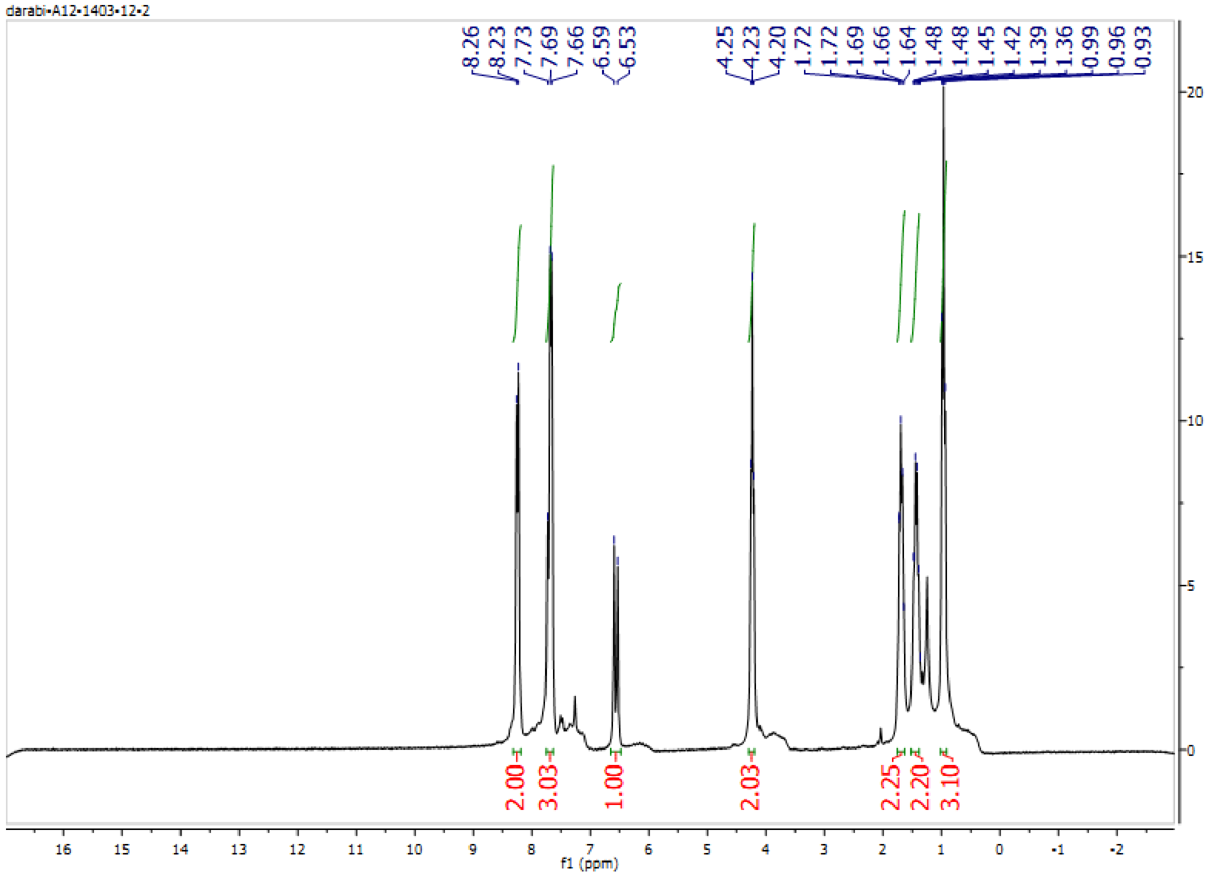

Supplement: NA-008-D5NA00951K-s001 [file NA-008-D5NA00951K-s001.pdf]
